# Manure substitution for chemical nitrogen enhances soil quality without compromising maize yield: a short-term field experiment in Northeast China

**DOI:** 10.3389/fpls.2025.1694608

**Published:** 2025-11-17

**Authors:** Chao Zhou, Jia Wang, Ting Xu, Kuan Pei, Baoxin Ma, Qingchao Li, Yang Liu, Xinying Ding, Yehui Han, Junqiang Wang

**Affiliations:** 1 Heilongjiang Academy of Agricultural Sciences, Qiqihar, China; 2 College of Agronomy, Heilongjiang Bayi Agricultural University, Daqing, Heilongjiang, China; 3 Tongliao Academy of Agricultural and Animal Husbandry Science, Tongliao, China; 4 Animal Husbandry and Veterinary Branch, Heilongjiang Academy of Agricultural Sciences, Qiqihar, China

**Keywords:** organic fertilizer application, maize yield, root traits, soil enzyme activity, soil bacterial community

## Abstract

**Introduction:**

Manure substitution for chemical nitrogen has the potential to enhance crop yield, improve soil quality, and reduce environmental risks. Soil microorganisms perform critical functions in mediating soil nutrient cycling after the organic manure application. Nonetheless, how organic manure substitution regulates microbial communities to influence soil quality and crop yield remains unclear.

**Methods:**

A one-year field experiment comprising four organic manure substitution rates (25%, 50%, 75%, and 100%) along with a no-substitution control was conducted.

**Results and disscussion:**

Compared to CK, only 25% substitution rate maintained maize yield, while 50‒100% manure substitution decreased maize yield by 15.9‒67.2%. This is primarily due to the decrease in root biomass (13.5‒29.1%), length (14.8‒43.3%), surface area (14.1‒48.8%) and volume (17.9‒53.4%). Manure substitution only increased soil quality index by 44‒55% in the 0-20 cm depth, mainly as a result of increased contents of soil organic C, total nitrogen, microbial biomass C and N, and enzyme activities. Moreover, manure substitution significantly increased the abundance of Actinobacteriota, Acidobacteriota, Gemmatimonadota, and Methylomirabiliota, with increases ranging from 12% to 101%. The strong correlations between these bacterial taxa and soil nutrient and C/N acquiring enzyme activities highlight their pivotal roles in boosting soil nutrients and enhancing soil quality. Therefore, organic manure substitution can be a sustainable fertilizer regime to enhance soil quality and maintain maize yield in Northeast China, and the optimal substitution rate is 25%.

## 1 Introduction

The black soil zone in Northeast China constitutes the country’s most crucial grain production base, which contributes over 30% of the national maize output (Wang et al., 2022). However, the overapplication of chemical fertilizers has accelerated the degradation of soil organic matter, leading to reduced nutrient availability (Tian et al., 2022), the disruption of soil structure (Ali et al., 2024), and impaired soil multifunctionality (Wang et al., 2024a). These adverse alterations in soil conditions threaten the stability of agricultural ecosystems and undermine the long-term viability of crop production systems (De Corato et al., 2024). Confronted with these challenges, preventing the degradation of black soils and maintaining their capacity for high and stable crop yields are essential for achieving sustainable agricultural development.

The application of organic manure represents an environmentally sustainable solution to mitigate soil degradation and support long-term agricultural productivity (Wu et al., 2025). China possesses substantial livestock manure resources; however, an estimated 20%–45% of this organic material remains unmanaged, resulting in high risks of environmental contamination and nutrient loss (Zhang et al., 2023). Integrating manure into agricultural systems presents a dual benefit: it enables efficient recycling of organic resources to enhance soil fertility while simultaneously mitigating environmental pollution caused by unmanaged agricultural waste (Yin et al., 2025). Nevertheless, existing studies indicate that the exclusive application of organic manure may fail to meet crop nutrient demands owing to its relatively low nutrient use efficiency (Li et al., 2022). Consequently, the partial substitution of chemical fertilizers with organic amendments has been proposed as a strategy to mitigate the limitations associated with sole manure application (Zhai et al., 2022). Niu et al. (2024) demonstrated that replacing 50% of synthetic fertilizer with manure was a sound option for achieving high crop yield and yield stability in the Guanzhong Plain. However, the effect of organic manure substitution to reduce synthetic nitrogen inputs in Northeast China remains inadequately evaluated.

Soil quality is a key indicator for assessing the capacity of soil to sustain ecosystem services (Salimani et al., 2025). Soil quality was assessed using an area-based method that integrated a set of biotic and abiotic indicators and was significantly influenced by fertilization management (Jia et al., 2022). Previous studies have indicated that incorporating organic amendments can improve soil quality and increase crop yields in various agricultural systems (Li et al., 2025). These benefits are largely attributed to improvements in soil nutrient availability, physical properties, and microbial ecological environment (Ju et al., 2022; Zhu et al., 2025). Sihi et al. (2017) observed that organic fertilizer applied in Sierozem soil can enhance soil water content and reduce soil bulk density compared to conventional mineral fertilization practices. Organic fertilizer can also increase soil quality by enhancing soil organic C, total N, and total P (Tian et al., 2022). At the same time, organic amendments can mitigate inorganic nitrogen leaching—specifically ammonium and nitrate—by enhancing the soil’s nutrient retention capacity (Guo et al., 2025). Soil enzyme activities are widely recognized as sensitive indicators of soil quality (Trasar-Cepeda et al., 2008). Organic amendments have been directly linked to increased activities of soil C- and N-related enzymes, including urease, invertase, catalase, and various hydrolytic enzymes (Feng et al., 2025). While most studies have integrated these indices to evaluate soil quality under organic fertilization applied (Jiang et al., 2022; Zhou et al., 2022), few have focused on the organic manure substitution for chemical N, particularly in the severely soil-degraded regions of Northeast China.

The soil microbiome serves as a fundamental driver of soil functionality and fertility, with its compositional structure and diversity being strongly influenced by fertilization management practices (Li et al., 2022; Yang et al., 2023). The partial application of organic amendments can effectively improve microbial habitat conditions and alter microbial community structure (Zhou et al., 2024). For example, long-term manure application has been proven to significantly increase the abundance of Proteobacteria and Chloroflexi in reddish paddy soil (Cui et al., 2018). Additionally, Liu et al. (2024) demonstrated that the combined application of manure and chemical fertilizers enhanced the abundance of bacterial taxa associated with nutrient cycling and utilization efficiency, particularly Firmicutes, Actinobacteria, and Planctomycetes. Shifts in soil microbial community structure and function are primarily driven by fertilizer-induced alterations in soil physicochemical properties (Ma et al., 2025). Consequently, the high sensitivity of microbial community properties to soil nutrient dynamics makes them reliable biomarkers for evaluating soil quality (Li et al., 2023). However, the integration of soil microbial indicators into soil quality assessments has rarely been evaluated under organic fertilizer substitution for chemical fertilizers.

Therefore, we carried out a one-year field experiment with different manure substitution rates for chemical fertilizer in the Northeast China Mollisol region to 1) evaluate the influence of organic manure substitution on soil physicochemical properties, enzyme activities, and soil quality; 2) identify the response of key soil microbial taxa to varying manure substitution rates; and 3) elucidate the microbial mechanisms through which organic fertilizer substitution influences maize yield and soil quality. We hypothesized that organic manure substitution would improve soil quality and crop yield by enhancing soil physicochemical properties and enzyme activities, as well as by enriching specific soil microbial taxa. To elucidate the microbial mechanisms by which the partial substitution of chemical fertilizers with organic manure influences soil quality and crop productivity, we conducted a comprehensive analysis that integrated soil physicochemical properties, enzyme activities, bacterial communities, and key root architecture traits, with ultimate crop yield. The goal of this experiment was to determine the most effective rate of organic manure replacement that both improves soil quality and maintains maize yield in the Northeast China Mollisol zone.

## 2 Materials and methods

### 2.1 Study site

The study was carried out at the Qiqihar Experimental Station, Heilongjiang Academy of Agricultural Sciences (47°36′ N, 123°65′ E; elevation 127 m), situated in Qiqihar City, Heilongjiang Province, China. The experimental site experiences a mid-temperate continental monsoon climate, with a mean annual precipitation of 434.5 mm and an average temperature of 4.7 °C. The soil type was a Chernozem Mollisol (IUSS Working Group WRB 2015). The topsoil layer properties were soil organic carbon (SOC) 21.9 g kg^−1^, alkaline hydrolyzed nitrogen (AHN) 99.8 mg kg^−1^, pH 8.4, and bulk density (BD) 1.58 g cm^−3^.

### 2.2 Experimental design

A randomized complete block design with three replicates (*n* = 3) was implemented in 2024. The experiment comprised five treatments: CK, 100% chemical fertilizer N; 25%, 25% dairy manure N substitution; 50%, 50% dairy manure N substitution; 75%, 75% dairy manure N substitution; and 100%, 100% dairy manure N substitution. The control (CK) treatment received fertilizer applications at rates of 187.5 kg N, 42.6 kg P, and 62.2 kg K per hectare, following local agronomic recommendations. Nutrient application rates for all treatments are detailed in **Table 1**. Maize (cv. ‘Nendan 22’) was planted at a density of 67,500 plants ha^−1^, with row spacing of 65 cm and plant spacing of 22 cm within rows. The crop residues were incorporated to a depth of 0–20 cm each year. To ensure seed germination, all plots were irrigated with 30 mm of water following maize sowing. Neither pesticides nor herbicides were applied throughout the experiment. All other field management practices aligned with those commonly employed in the region.

**Table 1:** Details of fertilizer treatments and fertilizer rates (kg ha^−1^ year^−1^).

Treatment	Chemical fertilizer			Manure			Total		
	N	P	K	N	P	K	N	P	K
CK	187	42.6	62.2	0	0	0	187	42.6	62.2
25%	140	42.6	62.2	47.0	0.42	1.46	187	43.0	63.7
50%	93.7	42.6	62.2	93.3	0.83	2.89	187	43.4	65.1
75%	46.8	42.6	62.2	140	1.25	4.35	187	43.9	66.6
100%	0	42.6	62.2	187	1.66	5.81	187	44.3	68.0

CK, 0% manure N substituted; 25%, 25% manure N substituted; 50%, 50% manure N substituted; 75%, 75% manure N substituted; 100%, 100% manure N substituted.

### 2.3 Soil sampling and analysis

Soil samples were obtained from depth intervals of 0–20 and 20–40 cm following maize harvest on 5 October 2024. Each soil sample was separated into three subsamples. One subsample was stored for analysis of SOC, total nitrogen (TN), available nitrogen (AHN), available phosphorus (AP), and available potassium (AK), following the analytical procedures described by Bao (2000). Soil BD was determined using the ring-knife sampler (volume of 100 cm^3^) method. Dissolved organic carbon (DOC) and nitrogen (DON) in the potassium sulfate extracts were measured using a carbon–nitrogen analyzer. The second subsample was kept at 4 °C to measure microbial biomass carbon (MBC)/microbial biomass nitrogen (MBN) and six hydrolyzing enzyme activities. The third subsample was stored at −20 °C for DNA extraction. Soil MBC and MBN were quantified using chloroform fumigation extraction (Vance et al., 1987). The activities of six hydrolyzing enzyme activities were identified using the fluorescence methods (Marx et al., 2001). Microbial metabolic limitations were also evaluated, and the calculation methods are according to (Equations 1–3):

(1) $$\begin{array}{c} \text{Vector length}=\\ \sqrt{{\left(\frac{BG+BX+CE}{BG+BX+CE+ACP}\right)}^{2}+{\left(\frac{BG+BX+CE}{BG+BX+CE+LAP+NAG}\right)}^{2}} \end{array}$$

(2) $$\begin{array}{c} \text{Vector angle}=\\ \text{Degrees}(\text{Atan}2(\frac{BG+BX+CE}{BG+BX+CE+ACP},\frac{BG+BX+CE}{BG+BX+CE+LAP+NAG})) \end{array}$$

Soil quality index (SQI) was calculated by comparing the area on a radar graph comprising all soil indicators (Feng et al., 2024).

(3) $$SQI_{-area}=0.5\cdot \sum_{i}^{n}SL_{i}^{2}\cdot \sin(\frac{2\pi }{n})$$

where *n* represents the number of soil indicators used for SQI calculation, and “ $SL_{i}$“ represents the linear score of the *i*th soil indicator.

### 2.4 Crop yield and root traits

Maize grain yield was quantified by harvesting within a 6-m^2^ area per plot, followed by air-drying and mechanical threshing to determine dry grain weight. Root traits were analyzed using an Epson Perfection V750 Pro scanner, and image analysis using the WinRHIZO software quantified root architectural parameters, including length, surface area, and volume. Root biomass was determined following oven-drying at 80°C.

### 2.5 DNA extraction and sequencing

DNA was extracted using the OMEGA Soil DNA Kit (M5635-02) (Omega Bio-Tek, Norcross, GA, USA) and kept at −20°C before further analysis. Samples were sequenced via Illumina^®^ MiSeq (Genesky Biotechnologies Inc., Shanghai, China). The V4–V5 hypervariable regions of the 16S rRNA gene were amplified with the primers 907R (5′-CCGTCAATTCMTTTRAGTTT-3′) and 515F (5′-GTGCCAGCMGCCGCGG-3′). DNA quality was assessed using a NanoDrop NC2000 spectrophotometer (Thermo Fisher Scientific, Waltham, MA, USA) for concentration and purity, supplemented by agarose gel electrophoresis for integrity verification.

### 2.6 Statistical analysis

The normality of all datasets was verified using the Shapiro–Wilk test. The influences of organic manure substitution on maize yield, root biomass, length, surface area, and volume were analyzed using one-way ANOVA. Two-way ANOVA was employed to analyze the effects of organic manure substitution and soil depth (0–20 and 20–40 cm), along with their interaction, on soil physicochemical properties, hydrolase activities, soil quality index, microbial diversity, and dominant bacterial taxa. The correlations between soil physicochemical properties, hydrolase activities, maize yield, and soil quality were also assessed using Mantel tests implemented with the “linkET” package. Meanwhile, key predictors of maize yield and soil quality were evaluated using a random forest model, executed using the “rfPermuta” package. All statistical analyses were performed, and data visualizations were generated using the R software (v.4.1.3; R Core Team, 2022).

## 3 Results

### 3.1 Maize yield and root traits

Compared to CK, only 25% manure substitution maintained maize yield, while 50%–100% substitution ratios significantly reduced maize yields by 15.9%–67.2% (**Table 2**; *p* < 0.05). Moreover, manure substitution treatments decreased root biomass, root length, root surface area, and volume by 13.5%–29.1%, 14.8%–43.3%, 14.1%–48.8%, and 17.9%–53.4% respectively (**Table 2**; *p* < 0.05).

**Table 2:** Effects of substituting chemical nitrogen with manure on maize yield and root traits.

Treatment	Yield (Mg ha^−1^)	Root biomass (g plant^−1^)	Root length (×10^3^ cm plant^−1^)	Root surface area (×10^2^ cm^2^ plant^−1^)	Root volume (cm^3^ plant^−1^)
CK	11.3 ± 0.83 a	19.2 ± 2.02 a	5.86 ± 0.86 a	13.9 ± 2.44 a	26.2 ± 5.63 a
25%	11.7 ± 1.46 ab	18.4 ± 0.97 a	5.12 ± 0.66 a	12.6 ± 1.69 ab	24.9 ± 3.75 a
50%	10.3 ± 0.69 b	15.5 ± 1.03 bc	4.99 ± 0.43 a	10.2 ± 1.44 bc	15.6 ± 1.88 a
75%	8.35 ± 1.22 c	16.4 ± 1.41 ab	4.92 ± 0.42 a	11.9 ± 1.93 ab	21.5 ± 3.28 a
100%	4.03 ± 0.35 d	13.5 ± 1.39 c	3.32 ± 0.34 b	7.09 ± 1.23 c	12.2 ± 3.38 a
One-way ANOVA					
Treatment	<0.001	0.005	0.004	0.008	0.004

Values are means ± standard deviations (*n* = 3). Different letters denote significant differences among treatments at 0.05 level.

CK, 0% manure N substituted; 25%, 25% manure N substituted; 50%, 50% manure N substituted; 75%, 75% manure N substituted; 100%, 100% manure N substituted.

### 3.2 Soil indices and quality index

**Figure 1:**
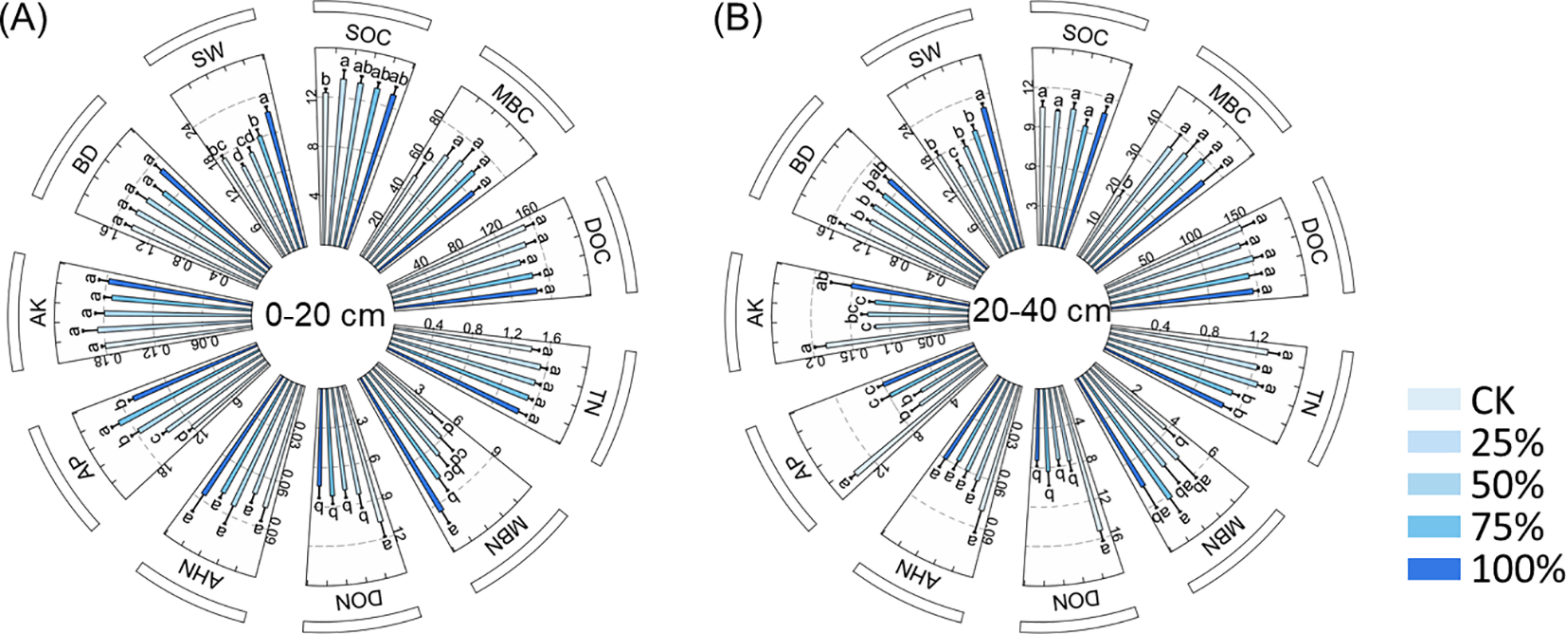
Effects of substituting chemical nitrogen with manure on soil physicochemical properties at depths of 0–20 **(A)** and 20–40 cm **(B)**. CK, 0% manure Nsubstituted; 25%, 25% manure N substituted; 50%, 50% manure N substituted; 75%, 75% manure N substituted; 100%, 100% manure N substituted.SOC, soil organic carbon; TN, total nitrogen; MBC, microbial biomass C; MBN, microbial biomass N; DOC, dissolved organic C; DON, dissolvedorganic N; AHN, alkaline nitrogen; AP, available phosphorus; AK, available potassium; BD, bulk density; SW, soil water content. Values are means ±standard deviations (n = 3). Different letters denote significant differences among treatments at 0.05 level.

Compared to CK, manure substitution increased the contents of SOC (6%–10%), MBC (28%–42%), MBN (32%–112%), AP (22%–59%), and SW (5%–22%), but decreased DON (23%–31%) in the 0–20-cm depth (**Figure 1A**; *p* < 0.05). In the 20–40-cm depth, MBC (92%–112%), MBN (28%–59%), and SW (6%–22%) were increased by manure substitution treatments, whereas TN (2%–17%), DON (45%–52%), AP (42%–60%), AK (17%–35%), and BD (8%–14%) declined compared to CK (**Figure 1B**; *p* < 0.05).

**Figure 2:**
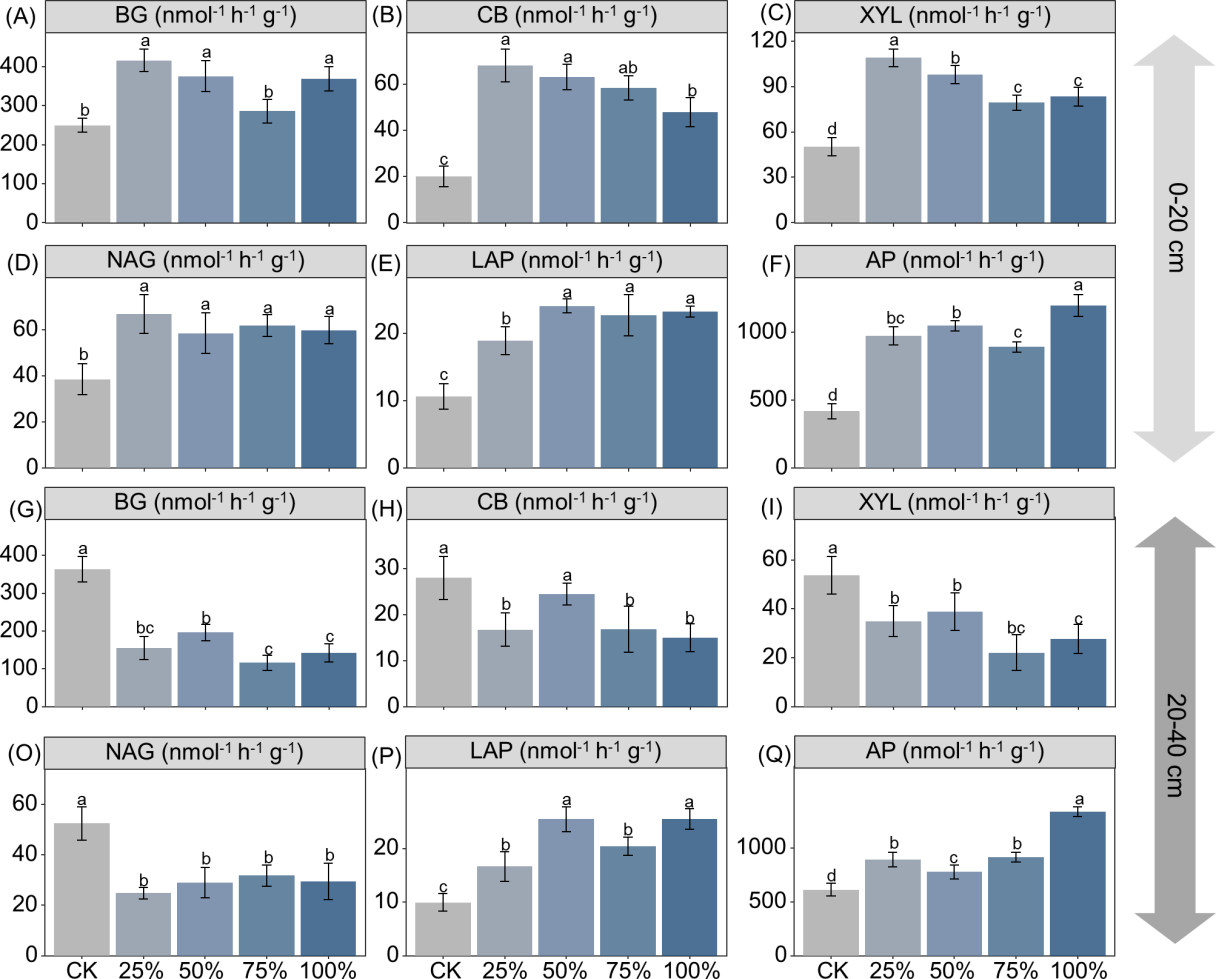
Effects of substituting chemical nitrogen with manure on soil C/N/P acquiring enzyme activities at depths of 0–20 **(A–F)** and 20–40 cm **(G–Q)**. CK, 0% manure N substituted; 25%, 25% manure N substituted; 50%, 50% manure Nsubstituted; 75%, 75% manure N substituted; 100%, 100% manure N substituted. BG, b-1,4-glucosidase; XYL, b-D-xylopyranoside; CB, b-D-cellobiosidase; NAG, b-1,4-N-acetylglucosaminidase; LAP, L-leucineaminopeptidase; ALP, acid phosphatase. Values are means ± standard deviations (n = 3). Different letters denote significant differences among treatments at 0.05 level.

**Figure 3:**
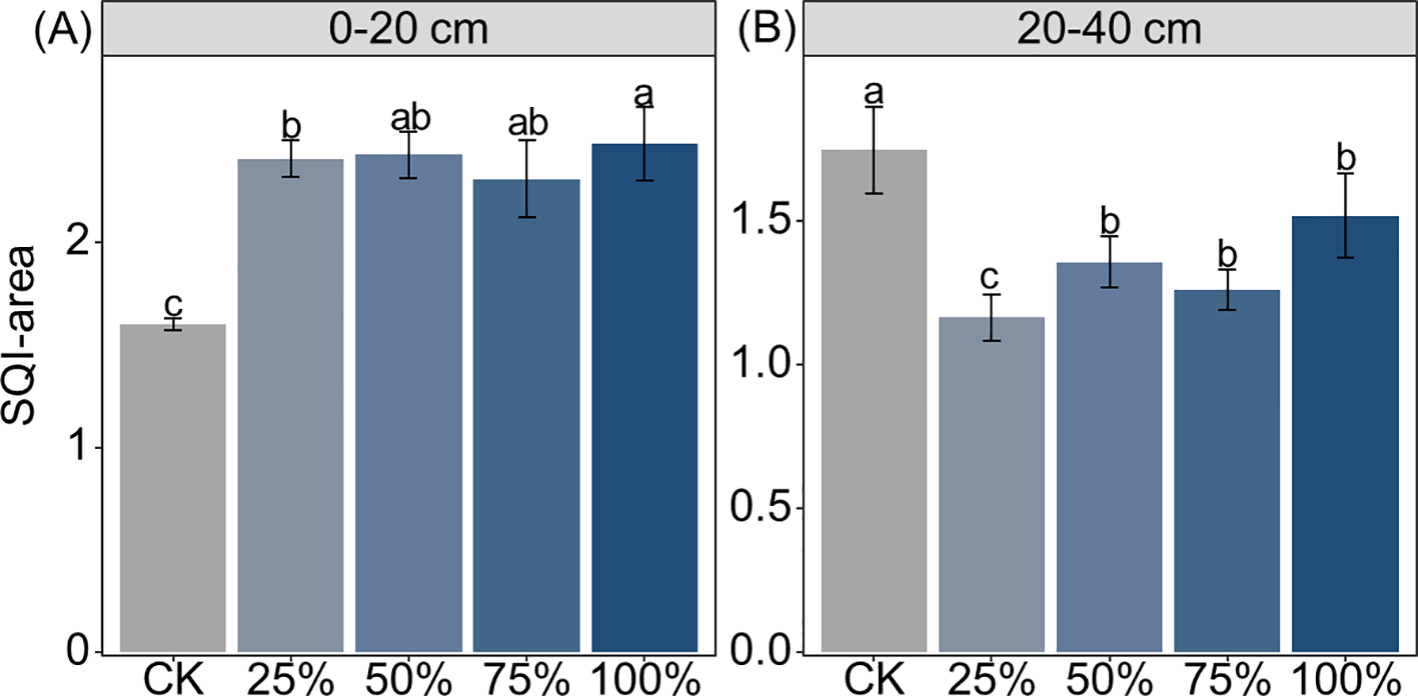
Effects of substituting chemical nitrogen with manure on soil quality index (SQI) area at depths of 0–20 **(A)** and 20–40 cm **(B)**. CK, 0% manure Nsubstituted; 25%, 25% manure N substituted; 50%, 50% manure N substituted; 75%, 75% manure N substituted; 100%, 100% manure N substituted.Different letters denote significant differences among treatments at 0.05 level.

Manure substitution increased the activities of β-1,4-glucosidase (BG), β-d-cellobiosidase (CB), β-d-xylopyranoside (XYL), β-1,4-*N*-acetylglucosaminidase (NAG), acid phosphatase (ALP), and l-leucine aminopeptidase (LAP) by 15%–67%, 138%–239%, 57.9%–117%, 52%–74%, 78%–125%, and 112%–185% in the 0–20-cm depth, respectively (**Figure 2**; *p* < 0.05). In contrast, manure substitution decreased the activities of BG (46%–68%), CB (12%–40%), XYL (28%–59%), and NAG (40%–53%), but increased LAP (67%–155%) and ALP (27%–118%) activities in the 20–40-cm depth (**Figure 2**; *p* < 0.05). Moreover, the SQI score increased by 44%–55% with manure substitution, but decreased by 13%–33% in the 20–40-cm depth (**Figure 3**; *p* < 0.05).

### 3.3 Soil bacterial community structure and composition

**Figure 4:**
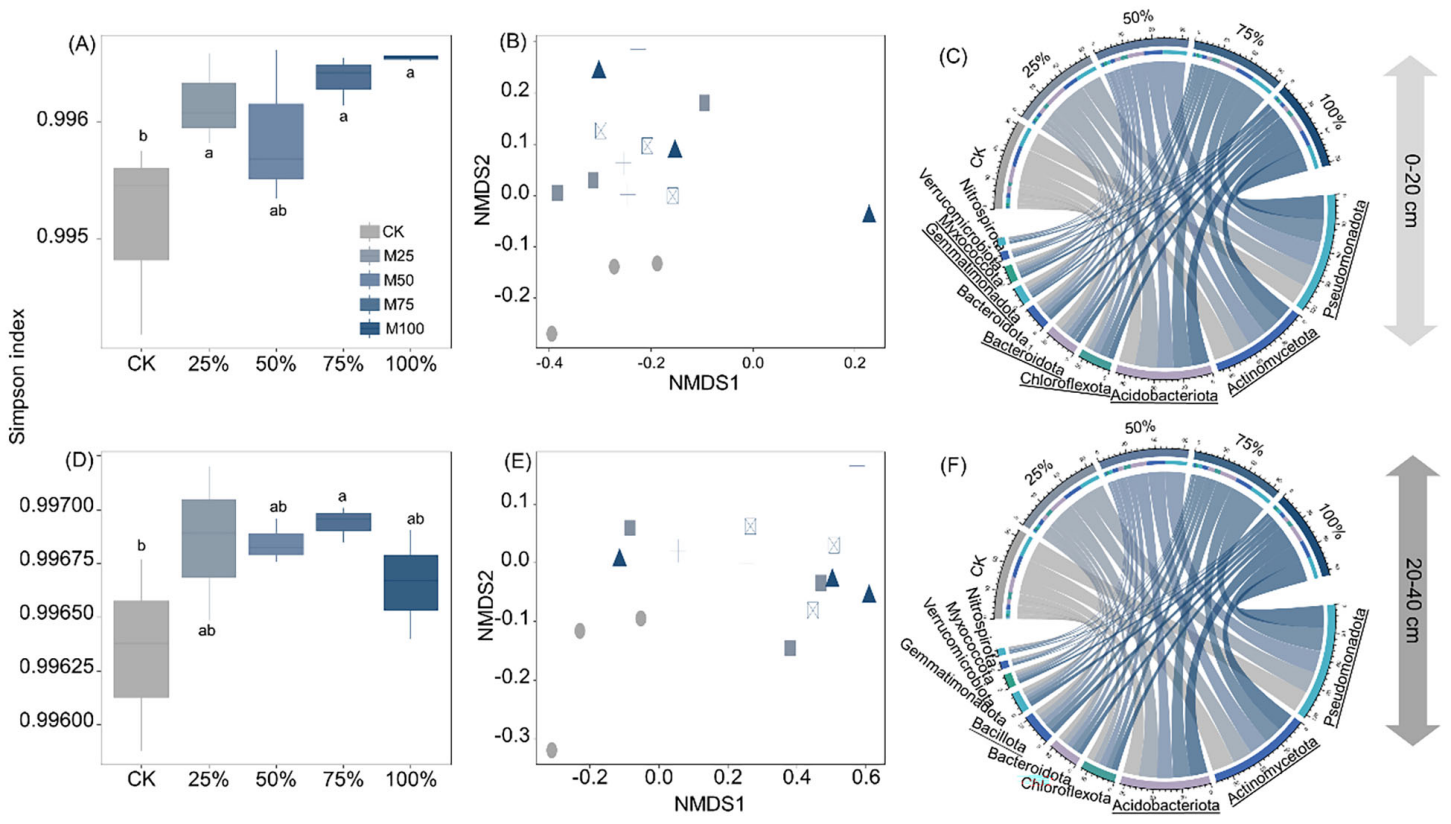
Effects of substituting chemical nitrogen with manure on bacterial diversity and community composition at depths of 0–20 **(A–C)** and 20–40 cm **(D–F)**. CK, 0% manure N substituted; 25%, 25% manure N substituted; 50%, 50% manure N substituted; 75%, 75% manure N substituted; 100%, 100% manure N substituted. Different letters and underlined notations denote significant differences among treatments at 0.05 level.

Compared to CK, manure substitution significantly increased the Simpson index of bacteria by 0.08%–0.14% in the 0–20-cm depth and 0.03%–0.06% in the 20–40-cm depth (**Figures 4A, D**; *p* < 0.05). Bacterial community structure was significantly altered by manure substitution treatments (**Figures 4B, E**).

In the 0–20-cm depth, manure substitution increased the relative abundance of Actinomycetota (14%–28%), Acidobacteriota (12%–35%), Gemmatimonadota (64%–88%), and Methylomirabilota (55%–101%) while decreasing Bacteroidota (21%–57%), Chloroflexota (15%–24%), and Pseudomonadota (13%–29%) (**Figure 4C**, *p* < 0.05). In the 20–40-cm depth, manure substitution increased the relative abundance of Pseudomonadota (5–6%), Acidobacteriota (11%–18%), and Bacillota (12%–21%), but decreased Actinomycetota (12%–20%) compared to CK (**Figure 4F**, *p* < 0.05).

### 3.4 Drivers of maize yield and soil quality index

**Figure 5:**
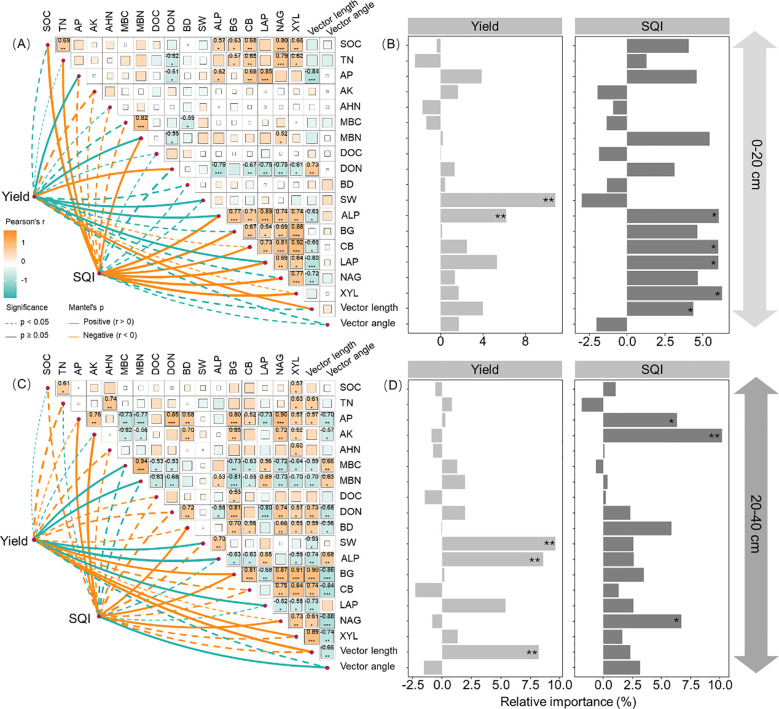
Correlations between soil physicochemical properties, enzyme activities, vector length, and angle affecting maize yield and soil quality index atdepths of 0–20 **(A, B)** and 20–40 cm **(C, D)**. SOC, soil organic C; TN, total nitrogen; AP, available phosphorus; AK, available potassium; AHN, alkaline hydrolyzedN; MBC, microbial biomass C; MBN, microbial biomass N; DOC, dissolved organic C; DON, dissolved organic N; BD, bulk density; SW, soil watercontent; BG, b-1,4-glucosidase; XYL, b-D-xylopyranoside; CB, b-D-cellobiosidase; NAG, b-1,4-N-acetylglucosaminidase; LAP, L-leucineaminopeptidase; ALP, acid phosphatase. ***p < 0.001; **p < 0.01; *p < 0.05.

**Figure 6:**
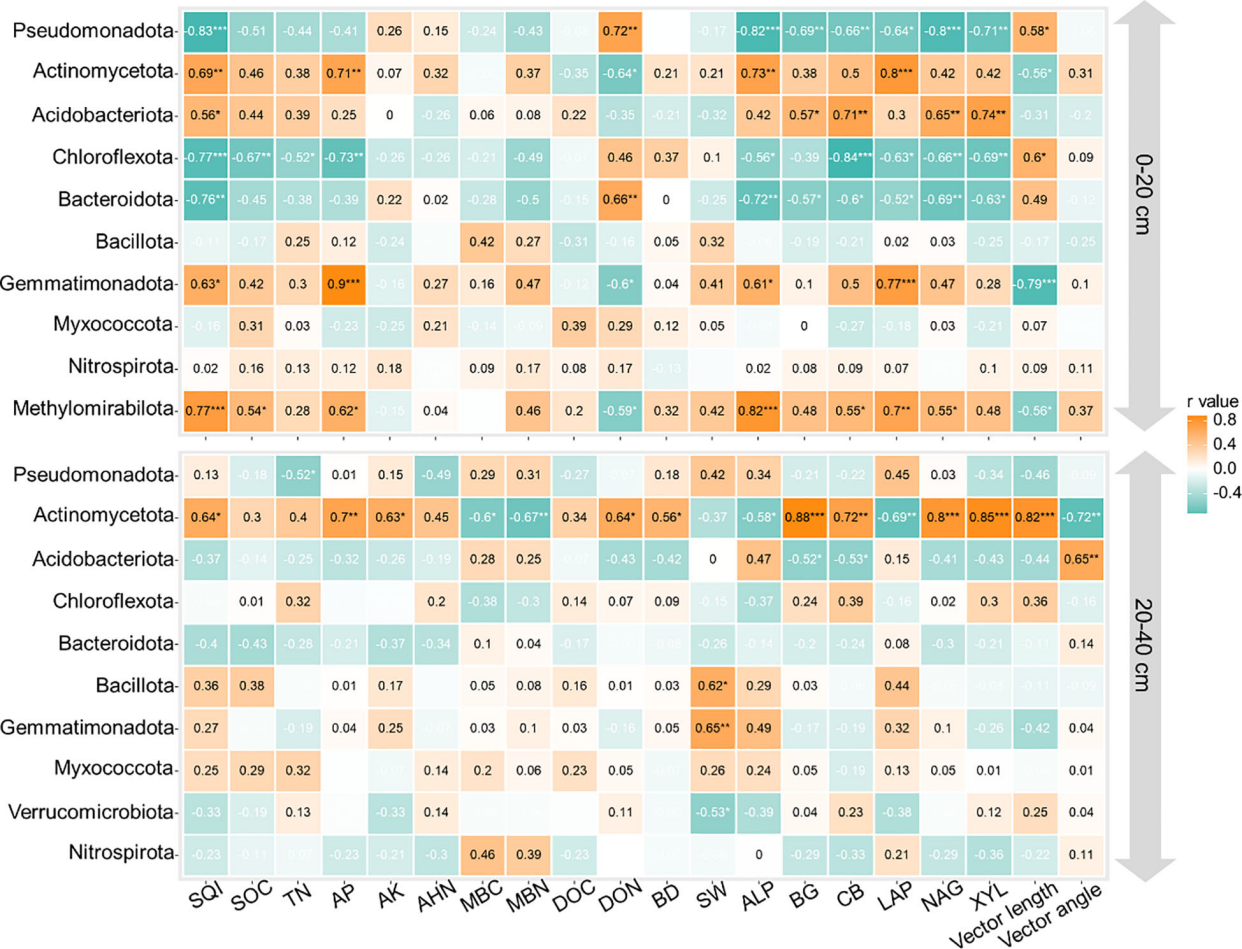
Correlations between the top 10 abundance of bacterial phyla and soil physicochemical properties, enzyme activities, vector length, and angle at depths of 0–20 and 20–40 cm. SQI, soil quality index; SOC, soil organic C; TN, total nitrogen; AP, available phosphorus; AK, available potassium; AHN, alkaline hydrolyzed N; MBC, microbial biomass C; MBN, microbial biomass N; DOC, dissolved organic C; DON, dissolved organic N; BD, bulk density; SW, soil water content; BG, β-1,4-glucosidase; XYL, β-d-xylopyranoside; CB, β-d-cellobiosidase; NAG, β-1,4-*N*-acetylglucosaminidase; LAP, l-leucine aminopeptidase; ALP, acid phosphatase. ***p < 0.001; ** *p* < 0.01; *p < 0.05.

Maize yield correlated with MBN, SW, ALP, LAP, and vector length at both depths of 0–20 and 20–40 cm, as well as with AP and DON in the 0–20-cm depth and with MBC, BG, and XYL in the 20–40-cm depth (**Figures 5A, C**; *p* < 0.05). Soil water content, ALP, and vector length were identified as the main factors influencing maize yield (**Figures 5B, D**; *p* < 0.05). These key factors are also linked to the relative abundance of Pseudomonadota, Actinomycetota, Gemmatimonadota, and Methylomirabilota (**Figure 6**; *p* < 0.05).

Soil quality index was correlated with SOC, TN, MBN, ALP, BG, CB, LAP, NAG, and XYL in the 0–20-cm depth and with AP, AK, BD, BG, NAG, and vector angle in the 20–40-cm depth (**Figures 5A, C**; *p* < 0.05). SQI was primarily affected by ALP, CB, LAP, XYL, and vector length in the 0–20-cm depth, while AP, AK, and NAG were more influential in the 20–40-cm depth (**Figures 5B,D**; *p* < 0.05). These key factors were also associated with the relative abundance of Actinomycetota, Acidobacteriota, Chloroflexota, and Methylomirabilota in the 0–20-cm depth and Actinomycetota in the 20–40-cm depth (**Figure 6**; *p* < 0.05).

## 4 Discussion

### 4.1 Effects of organic manure substitution with chemical N on maize yield

Contrary to our hypothesis, organic manure substitution negatively affected maize yield, with greater reductions observed at higher substitution rates (**Table 2**). These results were consistent with the results of Niu et al. (2024), who found that 75% substitution and above markedly dropped the annual yield of winter wheat and summer maize. This may be due to the excessive proportion of manure substitution causing insufficient N supply, as its slow-release nature requires time to mineralize (**Figure 1**; Wang et al., 2020). The lower DON in the 0–20-cm and 20–40-cm layers and its positive correlation with maize yield could support this point (**Figures 1**, **5A**). Inhibited maize root growth under a high manure substitution rate served as another crucial factor impairing yield formation (**Table 2**; Wang et al., 2023). In this study, 50%–100% substitution rates significantly decreased root biomass, length, surface area, and volume (**Table 2**). This will undoubtedly restrict the nutrient uptake capacity of maize, consequently compromising its development and reducing yield (Feng et al., 2024). Interestingly, we observed that the 25% substitution rate could maintain the maize root growth and yield formation compared with the CK treatment. This can be attributed to the effective synchronization of nitrogen supply and crop demand: the 75% inorganic fertilizer readily satisfied the early peak demand, while the subsequent slow mineralization of the 25% organic manure complemented nitrogen availability during the later reproductive growth stages, thus ensuring stable yield (**Table 2**, Wang et al., 2020). Collectively, substitution chemical fertilizers with organic manure failed to enhance maize yield in northeast China, but a 25% substitution rate could serve as a sustainable fertilization strategy to reduce chemical fertilizer without a yield penalty.

### 4.2 Effects of organic manure substitution with chemical N on soil quality

Consistent with our hypothesis, the substitution of chemical fertilizer with organic manure significantly affected soil quality, but exerted distinct effects between depths of 0–20 and 20–40 cm. For the 0–20-cm depth, manure substitution increased soil quality by effectively improving soil chemical properties (e.g., SOC, TN, MBC, MBN, AP, and soil water content). The results may be because 1) the application of organic manure drives the soil carbon cycle by facilitating the transformation of organic matter, thereby increasing soil quality (Feng et al., 2024). 2) Manure substitution treatments promote nutrient release from organic manure and reduce nutrient losses (Cui et al., 2020). For example, organic manure can reduce ammonia volatilization and nitrate leaching through adsorption and, thereby, indirectly increase nutrient levels in the upper layer and contribute to soil quality improvement (Wang et al., 2024b). 3) The application of organic manure can accelerate soil C and N cycling via microbial-mediated priming effect, which may increase soil nutrient availability and improve soil quality (Zhou et al., 2025). These results can be further supported by the high content of microbial biomass C/N and its related enzyme activities (**Figures 1**, **2**). It should be noted that the manure substitution treatments supplied substantial inputs of AP and AK relative to CK, which was also a significant contributor to topsoil quality.

However, the soil quality in the 20–40-cm depth under manure substitution treatments exhibited an opposite trend, being significantly reduced compared to the CK. This can be attributed to three main reasons: first, since both organic and chemical fertilizers were primarily incorporated into the topsoil through rotary tillage, manure substitution treatments had limited influence on the deeper soil layers (He et al., 2022). Second, organic manure input reduced the leaching of available nitrogen through physical adsorption (Wei et al., 2021). This can also be supported by the lower contents of TN, DON, and AHN in the 20–40-cm depth (**Figure 1B**). Third, high rates of manure-substituted chemical fertilizer inhibited maize root growth due to insufficient N supply. This likely led to diminished root residue incorporation into deeper soil layers, consequently reducing nutrient content and being detrimental to soil quality (Feng et al., 2024). Interestingly, we did not detect a significant association between the maize yield and soil quality. This is because a significant relationship between crop yield and soil quality in previous studies often emerges over longer time scales (He et al., 2022; Nie et al., 2025). In the short term, yield is predominantly influenced by immediate nutrient availability, while the benefits of soil quality improvements take longer to manifest and affect crop yield (Wang et al., 2020). 

### 4.3 Soil microorganism response to organic manure substitution with chemical N

Soil bacteria serve as critical mediators in soil nutrient cycling, and their diversity and community structure are linked to the soil quality and crop performance (Philippot et al., 2024). Our study found that manure substitution treatments significantly increased the Simpson index of soil bacteria at depths of 0–20 and 20–40 cm (**Figure 4**). In line with Han et al. (2021), the organic manure substitution with chemical N fertilizer enhanced soil bacterial α-diversity. This increase in bacterial diversity may be attributed to two reasons: first, the organic manure application supplied a broader spectrum of substrates that support the diverse bacterial community growth (Lin et al., 2019). Second, organic manure enables the direct introduction of its inherent microbial community into the soil, thereby contributing to an increase in bacterial diversity (Li et al., 2022). Interestingly, although the majority of organic fertilizer was concentrated in the 0–20-cm depth, bacterial diversity also increased significantly in the deeper layer. This phenomenon can be primarily attributed to the leaching of soluble organic compounds derived from organic fertilizer decomposition, which may activate a more dormant microbial community in the deeper layer (Chen et al., 2024). In addition, the vertical transport of bacteria from the upper layer may have contributed substantially to the enhanced bacterial diversity observed in the 20–40-cm depth (He et al., 2023). This process was likely facilitated by the increased soil water content resulting from organic manure application, which improved hydrological connectivity and enhanced bacterial mobility through the soil profile.

Manure substitution shifted the soil microbial community structure and boosted the prevalence of beneficial taxa (**Figure 4**). For example, our study observed that manure substitution treatments significantly enriched the relative abundance of Actinomycetota, Acidobacteriota, Gemmatimonadota, and Methylomirabilota compared to CK (**Figure 4**). These findings are consistent with the previous literature on the effects of organic manure application on soil microbial communities (Ji et al., 2018; Lu et al., 2023). Acidobacteriota play a pivotal role in nutrient cycling by secreting enzymes that hydrolyze recalcitrant organic matter into bioavailable forms (Feng et al., 2024). This point was evidenced by the significant correlation between Acidobacteriota and soil C/N-acq enzyme activities (**Figure 6**). Actinobacteria are recognized for their significant contribution to soil carbon cycling, primarily due to their pronounced ability to decompose recalcitrant polymeric compounds (Nie et al., 2025). The strong correlation between Actinobacteria and soil C- and N-related enzyme activities further evidenced this point. Collectively, the enrichment of key soil microorganisms driven by organic manure substitution accelerated soil nutrient turnover, thereby enhancing soil quality.

## 5 Conclusion

In summary, this study demonstrates that short-term organic manure substitution generally decreased maize yield, which can be largely attributed to suppressed root growth caused by limited nitrogen availability. Only 25% substitution rates maintained maize yield compared to 100% chemical fertilizer applied. In contrast, organic manure application significantly enhanced soil quality within the 0–20-cm layer. Random forest analysis identified the key factors affecting soil quality, including alkaline phosphatase, cellobiohydrolase, leucine aminopeptidase, xylosidase, and enzyme vector length. The increased abundance of Actinomycetota, Acidobacteriota, and Methylomirabilota, along with their positive correlations with C- and N-acquiring enzyme activities, suggests that organic manure promotes soil organic carbon accumulation and nutrient availability through the enrichment of beneficial microbial taxa. However, a decline in soil quality was observed in the 20–40-cm layer, likely associated with reduced available phosphorus, available potassium, and *N*-acetylglucosaminidase activity. Collectively, these findings suggest that 25% organic manure substitution can serve as a sustainable fertilization strategy in Northeast China, capable of maintaining crop productivity while enhancing topsoil quality.

## Data Availability

The datasets presented in this study can be found in online repositories. The names of the repository/repositories and accession number(s) can be found in the article/**Supplementary Material**.
